# Periodontal Disease in Dogs From Mexico: Description of Most Commonly Affected Teeth and Associated Factors

**DOI:** 10.1155/vmi/6628061

**Published:** 2025-02-28

**Authors:** Ana G. Villegas-Ferre, Eduardo Gutierrez-Blanco, Víctor M. Martínez-Aguilar, Gandhi S. Hernandez-Chan, Matilde Jiménez-Coello, Antonio Ortega-Pacheco

**Affiliations:** ^1^Department of Animal Health and Preventive Medicine, Faculty of Veterinary Medicine, Autonomous University of Yucatan, Km 15.5 Carretera Merida-Xmatkuil, Apdo. Postal 4-116 Itzimna, Merida, Yucatan, Mexico; ^2^Department of Periodontics, Faculty of Odontology, Autonomous University of Yucatan, C.61A x Av. Itzaes, Merida, Yucatan, Mexico; ^3^National Geointelligence Laboratory (GeoINT), Scientífic and Technological Park, Yucatan Carretera Sierra Papacal-Chuburná Pto. Km 5 Sierra Papacal, Merida C.P. 97302, Yucatan, Mexico; ^4^Laboratory of Microbiology, Regional Research Center “Dr. Hideyo Noguchi”, Autonomous University of Yucatan, Avenida Itzáes, No. 490 x Calle 59, Col. Centro, Merida C.P. 97000, Yucatan, Mexico

## Abstract

With the objective to evaluate the prevalence of periodontal disease (PD) and monitor the most affected teeth in dogs under the subtropical conditions in Mexico, 184 randomly selected dogs underwent a periodontal examination. Of the evaluated dogs, 78.8% showed some degree of gingivitis most of them (76.6%) with a moderate index, and 30.4% presented some degree of PD being most of them of a mild degree. Old age and small size dogs were more prone to develop PD as reported elsewhere. Higher mobility index and furcation were seen in maxillary teeth 108 and 208 and mandibular teeth 308 and 408, but some other teeth were involved in less proportion. On probing, bleeding was observed in maxillary teeth 202, 11, 102, and 202 and mandibular teeth 309 and 409. Dental plaque and calculus were more frequent in maxillary teeth 202 and 102 and mandibular teeth 309, 310, and 409. Finally, dental loss was observed with more frequency in all maxillary incisors (102, 201, and 202) and molars 109 and 209; mandibular molars 411 and 311 were more prompt to be losed. This study demonstrates the high prevalence of gingivitis and development of PD in dogs in subtropics in Mexico and reveals the predisposition of some maxillary and mandibular teeth to develop PD and consequently their loss. The clinical implications of the study indicate that special attention should be paid to these teeth to be checked when the dogs come for consultation, during brushing and in dental prophylaxis.

## 1. Introduction

Periodontal disease (PD) is a very common infectious-inflammatory pathology in dogs in which two different stages occur: gingivitis and periodontitis [1]. The prevalence of PD in dogs can reach up to 90% [2], and it can be detected from the age of three years [3, 4]. The evolution of this disease varies depending on various factors (i.e., there is a positive correlation between PD severity and age) [4]. PD may also be more frequent at earlier stages in small breeds due to a reduction in the size of their jaw and the grouping of their teeth [4, 5]. The physical consistency of food may be closely linked to the development of dental plaque, with more tendency to occur in animals eating soft food; food accumulation predisposes to the presence of plaque and gingivitis develops, and consequently a series of events are triggered that end in PD [6–9]. However, large epidemiological surveys have not demonstrated the advantages and reduced predisposition to PD in dogs consuming dry food [9]. PD can be classified into several stages according to clinical signs and severity of the lesions, ranging from simple gingivitis to plaque formation, gingival recession, inflammation, and destruction of alveolar bone with tooth loss [2]. Despite its high frequency, in Mexico, there is a lack of studies that investigate PD in domesticated dogs and factors that may be associated and which teeth are at greatest risk of developing this pathology, so the purpose of this study was to estimate the prevalence of PD in domestic dogs from Yucatan, Mexico, and determine the predisposition of the teeth to be more affected and the association with some factors.

## 2. Materials and Methods

### 2.1. Study Area

This was a cross-sectional study that was carried out in the city of Merida, located on the Yucatán Peninsula (19° 30′ and 21° 35′ North latitude; 87° 30′ and 90° 24′ West longitude), Mexico, characterized by a subhumid tropical climate, with rain during the months of May to November. Canine patients from various private veterinary clinics in the city and dogs referred to the Medical Education Unit (UEM) of the Faculty of Veterinary Medicine and Zootechnics (FMVZ) of the Autonomous University of Yucatán (UADY) were included in the study.

### 2.2. Sample Size and Inclusion Criteria

The sample size was determined by convenience, and at least 140 randomly selected animals were considered. Dogs older than 1 year of age, with no history of dental cleaning, of any age, breed, and sex, that came for consultation or sterilization campaigns carried out during the study period in the city of Mérida were included. All dog owners were informed about the aims of the study and the manipulation of dogs, and if agreed they signed an informed consent form. The study protocol was evaluated and accepted by the bioethics committee from the CCBA-UADY (CB-CCBA-2021-006).

### 2.3. Periodontal Examination

To estimate the frequency and degree of PD, a periodontal examination was performed on each of the dogs. A patient with PD was considered one who presented inflammation of the gums and tooth support tissues (alveolar bone and periodontal ligaments) and/or destruction of these [6]. Likewise, to confirm PD, radiographies were taken from each patient, using intraoral techniques such as periapical projection, which helps us to observe and analyze the entire tooth, including the crown of the teeth, its root, the apex, the surrounding bone tissue, and the periodontal space, to diagnose possible lesions that are not visible to the naked eye. For this, a portable X-ray equipment, model X-12l, with a tube voltage of 60 kw was used, including a focusing tube of 0.7 × 0.7 mm; the exposure time was 1-2 s and frequency used was 30 khz (kilohertz). For the periodontal examination, animals were anesthetized, using a dose of 7.5–25 mg/kg intramuscularly, of tiletamine-zolazepam (Zoletil Lab. Virbac). During the periodontal examination, the degree of gingivitis was determined, and the indices and criteria established [2] were used; for this, the four regions of the gum were evaluated: distal, mesial, buccal, and lingual of each dog's tooth. Probing of the gingival margins and their depth was performed using a simple periodontal probe model 582, MEDESY brand. The distance from the gingival margin to the end of the periodontal pocket was measured at three points on the surface of each tooth (surface, buccal, and lingual). Only pockets whose depth was greater than 3 mm were recorded, and the presence of bleeding or not was noted, bacterial plaque and/or dental calculus, tooth loss, exposure of the furcation and mobility was also recorded. For this evaluation, a periodontogram was used, adapting the one described [10] and using the periodontal indices used indicated [7] which were divided according to their degree of affection, in P0: null, P1: mild, P2: moderate and P3: severe. P1 (Characterized by gingivitis without loss of gingiva-tooth adhesion, normal periodontal probing and no bone loss on radiography), P2 (moderate periodontitis characterized by 25%–50% loss of adhesion, added to the signs of stage 1, furcation exposure, along with a PPD between 4 and 6 mm deep) and P3 (Advanced Periodontitis, where > 50% loss of attachment or bifurcation where exposure can be observed, as well as a PPD with at least 6 mm depth).

### 2.4. Statistical Analysis

The prevalence of PD (gingivitis and periodontitis) was determined by age (1–3 years, 4–6 years, and < 7 years), size (small, medium, and large) and sex (male or female) of the patients. A univariate analysis (chi-square test) was performed to determine the association between the variables studied and the degree of PD disease using GraphPad Prism statistical software. In cases where there were cells with less than 5 observations, Monte Carlo simulation statistical test was used to obtain a more precise *p* value.

## 3. Results

The periodontal examination was performed on 184 dogs, of which 115 were males and 69 were females; 73 dogs were small in size, 92 were medium in size, and 19 were large in size. Ages 1–3 years included 69 dogs, 4–6 years 61 dogs, and > 7 years 54 dogs.

### 3.1. Gingival Index (GI)

78.8% of the dogs evaluated showed some degree of gingivitis. Of these, 21.2% (*n* = 39) showed normal gum, 76.6% (*n* = 138) mild inflammation, and the other 2.2% (*n* = 7) moderate inflammation. No cases with severe gingivitis were found (Table 1).

### 3.2. Periodontal Index (PI)

Of the 184 dogs evaluated, 30.4% presented some degree of PD; 69.5% (*n* = 128) showed a null PI, 23.4% (*n* = 43) a mild PI, 3.8% (*n* = 7) a moderate PI, and 3.3% (*n* = 6) developed severe PI (Table 1).

### 3.3. Risk Factors

Table 2 shows the frequency of the GI in the 184 evaluated dogs. As seen, the mild GI is most commonly seen despite the age, size, and gender of dogs.

Table 3 shows the frequency of the PI in the dogs evaluated and the associated risk actors. Dogs > 7 years old had a greater predisposition (*p* < 0.05) to present PI1. Although a higher frequency of PI1 was found in female and large dogs, this was not significant.

### 3.4. Other Findings

#### 3.4.1. Dental Mobility

The teeth that presented the highest mobility index were the maxillary molars with dental formulas 108, 109, and 106, and on the right side, the most affected were 206, 207, and 208; the incisors also showed a higher dental mobility index, being 102 and 202 the most affected; in the mandibular teeth, the incisors are the ones that show the greatest number of affected teeth, with 401 and 301 being the teeth with the highest degree of affectation; as for the molars on the left side, the tooth with the highest degree of mobility was 408 and 308 on the opposite side.

#### 3.4.2. Furcation Exposure Index

**Figure 1:**
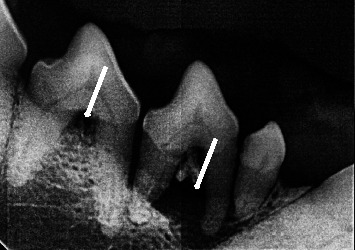
An example of furcation exposure in mandibular molars. Arrows show the normal furcation exposure.

The maxillary molars with the highest furcation exposure were those with dental formulas 108, 107, 207, and 208 (Figure 1). The highest exposure index was on the left side, with 10 pieces with an index of severe furcation exposure, 4 teeth with moderate exposure, and 1 piece with mild exposure. In the mandibular side, the teeth with the greatest exposure were 307, 308, 407, and 408, having 6 molars with severe furcation exposure and 5 pieces with moderate exposure. Radiographic evidence is seen in Figure 1.

#### 3.4.3. Dental Bleeding

**Figure 2:**
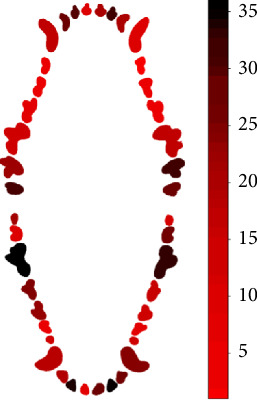
Heat map of dental bleeding from 184 evaluated dogs.

During probing, the maxilar molars show the greatest number of teeth with bleeding, particularly teeth 210 and 111, and incisors 102 and 202. For the mandibular teeth, the molars, both left and right, were the ones that show the greatest number of bleeding on probing; molars 409 and 309 are the most affected (Figure 2).

#### 3.4.4. Dental Plaque

**Figure 3:**
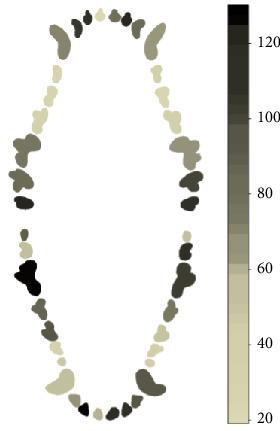
Heat map of dental plaque from 184 evaluated dogs.

In the case of dental plaque, the maxilar molars were more affected with this condition, being teeth 210, 211, and 111, the most frequently affected teeth; incisive teeth more affected were 101,102, and 202. In the case of the mandibular teeth, the pieces with most plaque were teeth 309, 310, 408, and 409 (Figure 3).

#### 3.4.5. Dental Calculus

**Figure 4:**
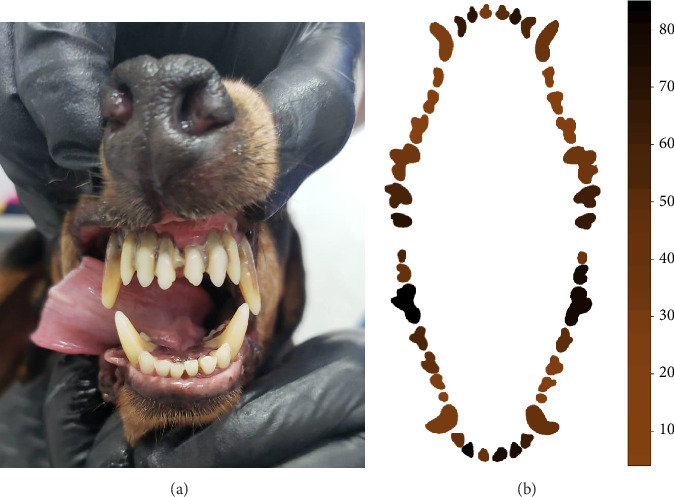
(a) The presence of dental calculus in the upper incisors and canines. (b) The heat map of dental calculus from 184 evaluated dogs.

Dental calculus was present most frequently in the maxilar incisives 102, 103, and 202 and molars 210 and 211. As for the mandibular teeth, the most affected pieces with the highest presence of calculus were molars 309, 310, and 409 and incisors 301 and 402 (Figure 4).

#### 3.4.6. Dental Losses

**Figure 5:**
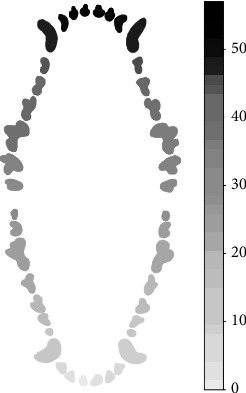
Heat map of dental losses from 184 evaluated dogs.

The incisive teeth of the maxilar side were more noticeable to be losed, particularly 101,102, 201, and 202 and the molars 119 and 209. For the mandibular teeth, molars 411 and 311 were the most commonly lossed (Figure 5).

## 4. Discussion

### 4.1. GI

Gingivitis is a reversible event of PD that is very frequently found in dogs [4, 11] as reported in the present study. However, in most cases, this lesion is mild, but it may progress to more severe stages. Food predisposes plaque accumulation that induces inflammation in the periodontium and induction of gingivitis. Its control by regular brushing prevents the progression and formation of plaque that eventually leads to PD. A significant positive relationship is reported between age and the severity of inflammation. Between 80% and 85% of dogs over 3 years of age may present some degree of gingival inflammation; however, in young animals, although less frequently, gingivitis to a lesser degree is commonly observed [12, 13]. In the present study, GI1 was very common in dogs of any age, size, or sex indicating the higher risk of developing PD in the conditions where the study was performed. Thus, veterinarian practitioners should recommend a dental prophylaxis program to prevent and treat gingivitis and PD.

### 4.2. PI

PD is a very common condition in dogs as a consequence of multiple factors triggering a cascade of events that result from inflammation of the gingiva to tooth loss. Food consumption and lack of dental care (dental prophylaxis, dental brushing, and chewing) are the predisposing factors leading to slow progressing events leading to gingivitis and PD. The prevalence found in the present study is high considering that the dogs were selected at random. In other studies, the prevalence of periodontitis varies between 10% and 20% [14, 15], but they were studies of dogs attending veterinary consultations which could have affected the results. More robust epidemiological studies show higher prevalence that can reach 44.0% or even 100% [2, 16]. As expected, small and older dogs are more likely to present higher rates and severity of PD, as has been described in other studies [11, 12, 17]. The prevalence of PD associated to the body size of dogs could be due to anatomical characteristics, such as skeleton and mouth size, since small dogs have proportionally longer teeth with less alveolar bone, smaller mouths, and teeth positioned with more closeness; this implies a greater contact surface between teeth and greater possibilities of plaque and calculus accumulation; in addition, small size dogs have less alveolar bone [18]. At old age, the predisposition of dogs to develop higher degrees of PD is more likely as already been reported [2–4, 19]. However, PD is a more complex and slow progressing problem involving several factors, not only size and age but also diet and natural predisposition of the dog. In this sense and because of all evidence generated, preventive measures are highly recommended to be established to all dogs.

### 4.3. Other Findings

The injuries associated with PD include various conditions such as dental motility, furcation exposure, dental bleeding, dental plaque, and dental calculus that ultimately end in dental losses. Additionally, bacteria present in the calculus of affected teeth may reach different tissues, and liberation of inflammatory factors, inducing systemic implications; systemic diseases such as chronic bronchitis, pulmonary fibrosis, endocarditis, interstitial nephritis, glomerulonephritis, and hepatitis [3, 20, 21].

Other lesions were found in a wide variety of maxillary and mandibular teeth. However, maxillary premolars 208 and 108 stood were more prone to mobility, furcation, bleeding, presence of plaque, development of calculus, and dental lost. Considering that the premolar and molar teeth of dogs are used for shearing, grinding, and chewing, they are more predisposed to presenting various pathologies, particularly the upper premolar teeth (208 and 108) and the first mandibular molars [22]. In the present study, it was precisely the maxillary premolars 208 and 108 and the mandibular molars 309 and 409 that presented the greatest lesions associated with PD.

The presence of these lesions is an important clinical finding because calculus results in the mineralization of dental plaque where an increase in the proportion of Gram-negative bacteria occurs as the inflammatory process (gingivitis) increases [7]. During this process, the epithelium attached to the tooth migrates apically, and the formation of the periodontal pocket occurs, the formations of highly vascularizated pockets that easily bleed when the dental probe is inserted [2]. In turn, periodontal pockets produce gingival recession, bone loss (horizontal and vertical), exposure of root surfaces (cementum), and tooth mobility in such a way that in advanced cases of PD, the furcation of multiple roots can be easily seen [23].

This finding demonstrates for the first time the predisposition of these teeth to suffer from PD and tooth loss, particularly of the molars and premolars. The accumulation of food in these teeth is due, in addition to the predisposition due to size and age, to the type of diet that dogs receive daily [24]. Dietary habits and dental care play an important role on the development of PD and eventually tooth loss; dry diets and lack of dental prophylaxis predispose the development of dental plaque, gingivitis, calculus accumulation, and damage of dental structures [5, 6].

These results point out the importance of reviewing these teeth more closely in settings such as during tooth brushing, dental prophylaxis, and routine clinical examinations. The use of dental chews has shown a positive effect by reducing gingivitis, dental plaque, and calculus due to its mechanical effect on the teeth, which in turn reduces the appearance of PD [1, 25, 26].

## 5. Conclusions

The results of this study indicate the high prevalence of gingivitis and development of PD in dogs under the conditions of dog ownership in the subtropics in Mexico; clinical findings such as dental plaque and calculus, motility, furcation, bleeding at probing, and dental losses are of special interest to the clinician indicating the degree of dental damage. Of special interest is the greater predisposition of some maxillary and mandibular teeth to develop PD and consequently their loss, which suggests that special attention should be paid to these teeth to be checked when the dogs come for consultation, during brushing and in dental prophylaxis.

## Figures and Tables

**Table 1:** Results of the gingival index (GI) and periodontal index (PI) in 184 dogs domiciled in the city of Merida, Yucatan, Mexico.

Gingival index *n* (%)				Periodontal index *n* (%)			
GI0	GI1	GI2	GI3	PI0	PI1	PI2	PI3
39 (21.2)	138 (76.6)	7 (2.2)	0 (0)	128 (69.5)	43 (23.4)	7 (3.8)	6 (3.3)

*Note:* GI0: normal. GI1: mild. GI2: moderate. GI3: severe. PI0: normal. PI1: mild. PI2: moderate. PI3: severe.

**Table 2:** Distribution of the gingival index (GI) in 184 dogs according to age, size, and gender.

	*n*	IG0	IG1	IG2	IG3
*Age*					
1–3	69 (37.5%)	28 (71.8%)	40 (29.0%)^a^	1 (14.3%)^a^	0 (0.0%)
4–6	61 (33.2%)	7 (17.9%)	52 (37.7%)^a^	2 (28.6%)^a^	0 (0.0%)
> 7	54 (29.3%)	4 (10.3%)	46 (33.3%)^a^	4 (57.1%)^a^	0 (0.0%)

*Size*					
Small	73 (40.0%)	21 (53.8%)	45 (32.6%)^a^	7 (100.0%)^a^	0 (0.0%)
Medium	92 (50.0%)	16 (41.0%)	76 (55.1%)^a^	0 (00.0%)^a^	0 (0.0%)
Large	19 (10.0%)	2 (5.2%)	17 (12.3.%)^a^	0 (0.0%)^a^	0 (0.0%)

*Gender*					
Female	69 (37.5%)	12 (30.8%)	54 (39.1%)^a^	3 (42.9%)^a^	0 (0.0%)
Male	115 (62.5%)	27 (69.2.%)	84 (60.9.%)^a^	4 (57.1%)^a^	0 (0.0%)

*Note:* Different letters in a column denote statistical difference (*p* < 0.05).

**Table 3:** Distribution of the periodontal index (PI) in 184 dogs according to age, size, and gender.

	*n*	PI0	PI1	PI2	PI3
*Age*					
1–3	69 (37.5%)	61 (47.7%)	8 (18.6%)^a^	0 (0.0%)^a^	0 (0.0%)^a^
4–6	61 (33.2%)	41 (32.0%)	16 (37.2%)^a^	3 (42.9%)^a^	2 (28.6%)^a^
> 7	54 (29.3%)	26 (20.3%)	19 (44.2%)^b^	4 (57.1%)^a^	5 (71.1%)^a^

*Size*					
Small	73 (40.0%)	44 (34.4%)	21 (48.8%)^b^	5 (71.4%)^a^	3 (42.9.0%)^a^
Medium	92 (50.0%)	69 (53.9%)	16 (37.2%)^a^	2 (28.6%)^a^	4 (57.1%)^a^
Large	19 (10.0%)	15 (11.7%)	6 (13.9%)^a^	0 (0.0%)^a^	0 (0.0%)^a^

*Gender*					
Female	69 (37.5%)	46 (35.9%)	19 (44.2%)^a^	2 (28.6%)^a^	3 (42.9%)^a^
Male	115 (62.5%)	82 (64.1%)	24 (55.8%)^a^	5 (71.4%)^a^	4 (57.1%)^a^

*Note:* Different letters in a column denote statistical difference (*p* < 0.05).

## Data Availability

The datasets used and/or analyzed during the current study can be obtained from the corresponding author upon request.
